# Editorial: DNA Vaccines

**DOI:** 10.3389/fmedt.2021.782986

**Published:** 2021-10-28

**Authors:** Ada Maria de Barcelos Alves, Silvia Beatriz Boscardin, Annie Elong Ngono

**Affiliations:** ^1^Laboratory of Biotechnology and Physiology of Viral Infections, Oswaldo Cruz Institute, Oswaldo Cruz Foundation, Rio de Janeiro, Brazil; ^2^Department of Parasitology, Institute of Biomedical Sciences, University of São Paulo, São Paulo, Brazil; ^3^Division of Inflammation Biology, La Jolla Institute for Allergy and Immunology, La Jolla, CA, United States

**Keywords:** DNA vaccines, Dengue virus, Zika virus, SARS-CoV-2, mycobacteria, emerging infectious disease, COVID-19

In the 1980's, DNA vaccines emerged as a promising revolution in the field of vaccines due to their effectiveness in the first trials with rodents and other small animals. In the following years, tests with non-human primates and human clinical trials dampened the initial enthusiasm for this vaccine approach, given the low immunogenicity observed after the inoculation of plasmid DNAs by conventional routes (1). One of the greatest obstacles was the delivery of the plasmid DNA. Nevertheless, electroporation or needle-free DNA inoculation brought a new perspective to these vaccines, with a significant increase in host cell transfections (2). In addition, nucleic acid-based immunizations (DNA or RNA) have several advantages compared to other vaccines. They are able to induce strong T cell responses and the *in vivo* antigen expression avoids incorrect folding which may influence specificity of the elicited antibodies (3).

Two articles in the present topic focused on the development of DNA vaccines against one of the most important arboviruses in the world, the Dengue virus (DENV). The first article (Alves et al.) showed results with the combination of three DNA vaccines encoding the DENV non-structural 1, 3, and 5 (NS1, NS3, and NS5). Immunization with the combined vaccines induced specific antibodies against the three proteins in C57BL/6 mice and production of IFN-γ. All vaccinated animals survived virus infection, while 80% of control mice succumbed. The work emphasizes the importance of the DENV non-structural proteins in protection, although the authors did not investigate the role of each DNA vaccine separately.

The second article on the present topic presented a detailed review of the DNA vaccines based on structural or non-structural DENV proteins (Alves et al.). The authors exposed the difficulties in the development of a vaccine against all the four dengue serotypes, without the risk of antibody-dependent enhancement of virus replication (ADE). They also discussed the use of DNA vaccines in combination with other vaccine strategies to induce more robust immune responses, especially of T cells. Combining different vaccines against the same pathogen is, in fact, a promising approach that is now also being used to fight SARS-CoV-2.

Pereira et al. investigated DNA vaccines based on the NS1 protein of another important arbovirus, the Zika virus (ZIKV). The authors constructed two plasmids encoding the full-length ZIKV NS1 protein and fused to the Herpes Simplex Virus glycoprotein D. The vaccines were evaluated in immunocompetent C57BL/6 mice and the results revealed that the plasmid encoding the chimeric gD1-NS1 induced better humoral and T cell responses with IFN-γ production. Immunodeficient AB6 (IFNAR1–/–) mice immunized with these plasmids were challenged with ZIKV and the gD1-NS1 based vaccine induced better protection. This work also emphasizes the potential of NS1 from different flaviviruses as protective antigens encoded by DNA vaccines.

Silva et al. presented a review of the use of plasmids encoding the Mycobacterial 65 heat shock protein (HSP65) as a therapeutic approach. The hsp65 DNA vaccine was shown to participate in the activation of innate and adaptive immunity. It modulates activation and cytokine release, specially IFN-γ, with promising potential to treat chronic or latent tuberculosis diseases, as well as multidrug resistant strains of *Mycobacterium tuberculosis*. Moreover, the action of this DNA vaccine goes far beyond *Mycobacterium* treatment. Its administration showed promising results in animal models for treatments against parasites and fungal infections, autoimmune diseases, allergic airway inflammation, and antitumor activity.

Xu et al. presented a review of recent clinical and preclinical studies with DNA vaccines targeting emerging infectious diseases. Another important characteristic of nucleic acid-based vaccines is that they can be designed and developed quickly, as long as the protein sequences of the pathogen are known, without the requirement of prior information on antigen production and purification. This feature is particularly important for emerging pathogens. Xu et al. reviewed data for DNA vaccines against Ebola, Zika, Venezuelan Equine Encephalitis, and Lassa Viruses. The vaccines against all these viruses were safe and well-tolerated in preclinical and/or clinical trials, and most of them induced humoral and cellular immune responses, with production of different cytokines.

Authors gave special attention to DNA vaccines against coronaviruses of human importance such as SARS-CoV, MERS-CoV, and SARS-CoV-2. Preclinical investigations with a DNA vaccine against SARS-CoV induced robust neutralizing antibodies and T cell responses, leading to a 6-fold lower lung viral load in mice after challenge (4). In the phase I trial, neutralizing antibodies were detected in 50% vaccinated individuals and all participants presented CD4+ T-cell responses. A DNA vaccine against MERS-CoV elicited potent humoral and cellular responses in different animal models, which correlate with protection after virus challenge. The vaccine was safe in the phase I trial and induced seroconversion in 94% of the individuals, with neutralizing antibody and T cell responses in 50% of them (5).

At the time of writing the article, no vaccine for mass immunization had been approved against SARS-CoV-2, which is responsible for the great pandemic of COVID-19 that we are experiencing today, with millions of deaths around the world. Since then, much has been learned about the virus and the disease. Xu et al. highlighted the importance of nucleic acid-based vaccines as a good inducers of T cell responses. Nowadays, there is more evidence of the importance of the cellular response to contain viral replication, which may explain the highest protection rates of the RNA vaccines against SARS-CoV-2 that are being administered in different countries (6). Moreover, different DNA vaccines were tested in animal models and some of them are currently in clinical trials^1^. Very recently, the first DNA vaccine for human use was approved in India against SARS-CoV-2. This fact will change the history of DNA vaccines and we will probably have more vaccines using this technology in the future.

## Author Contributions

AA wrote the article. SB reviewed the article. AE reviewed the article. All authors contributed to the article and approved the submitted version.

## Funding

This work was supported by the Carlos Chagas Filho Foundation for Research Support of the State of Rio de Janeiro (FAPERJ) (Grant Number: E-26/010.001959/2019), the National Institute of Science and Technology in Vaccines (INCTV) (Grant Number: 573547/2013), the Coordination of Improvement of Higher Education Personnel (CAPES) (Grant Number: 88882.332560/2019-01), and the Brazilian National Research Council (CNPq) (Grant Number: 310361/2019-2).

## Conflict of Interest

The authors declare that the research was conducted in the absence of any commercial or financial relationships that could be construed as a potential conflict of interest.

## Publisher's Note

All claims expressed in this article are solely those of the authors and do not necessarily represent those of their affiliated organizations, or those of the publisher, the editors and the reviewers. Any product that may be evaluated in this article, or claim that may be made by its manufacturer, is not guaranteed or endorsed by the publisher.

## Footnotes

^1^WHO. (2021). https://www.clinicaltrials.gov/ct2/results?term=DNA+vaccine&cond=vaccines+COVID-19.
